# Evaluating the Risk of Traumatic Brain Injury in Adults Following a Fall With Concomitant Use of Anti-Coagulation: A Retrospective Cohort Study

**DOI:** 10.7759/cureus.72629

**Published:** 2024-10-29

**Authors:** Aby Lal, Aneena Varghese, Sukhvinder Digpal

**Affiliations:** 1 General Medicine, Portsmouth Hospitals University National Health Service (NHS) Trust, Portsmouth, GBR; 2 General Internal Medicine, Portsmouth Hospitals University National Health Service (NHS) Trust, Portsmouth, GBR; 3 Internal Medicine, Portsmouth Hospitals University National Health Service (NHS) Trust, Portsmouth, GBR

**Keywords:** clinical frailty score, ct scan head, direct oral anticoagulants (doac), fall injury, geriatric fall, intra-cranial bleed, prophylactic and therapeutic anticoagulation, prophylactic anticoagulation, traumatic subarachnoid hemorrhage

## Abstract

Introduction

Falls and fractures are major health challenges for older adults in England. Despite the advantages of anticoagulants, their use in the elderly is often restricted due to concerns about fall-related injuries. However, there is a lack of clear data on the risk of discontinuing anticoagulation therapy solely due to fall risk. NICE guidelines (2021) advise that anticoagulation should not be withheld solely based on age or fall risk. This study aims to assess the incidence of significant brain injuries or intracranial haemorrhages in patients on anticoagulant therapy, also comparing the independent risk factors for traumatic brain injury.

Objective

This study aims to assess the incidence of TBI following falls in patients on anticoagulant therapy, comparing outcomes between those using DOACs and Vitamin K antagonists.

Methods

A retrospective cohort study was conducted at Queen Alexandra Hospital, University Hospitals Portsmouth NHS Trust, from November 2023 to May 2024. Data were collected from 3,468 CT head scans performed on patients with a history of falls, including 801 on anticoagulation therapy.

Results

Of the 801 patients on anticoagulation, 763 (95.2%) were aged 65 or older, with a mean age of 83.1 years. Acute hemorrhage was detected in 3.1% (25/801) of patients. Patients on Warfarin and Dabigatran had significantly higher TBI risk compared to those on Apixaban (6.7%, p=0.02; and 7.6%, p=0.01, respectively), while Edoxaban and Rivaroxaban showed no significant difference. Also, older age (≥65 years) and higher frailty scores (CFS 6 and 7) were associated with increased TBI risk (p<0.05). All patients with acute hemorrhage received conservative management, and two patients experienced mortality within six months.

Discussion

The study indicates that the risk of TBI following falls in anticoagulated patients is 3.1% relatively low, aligning with existing literature. This underscores the need for careful consideration before discontinuing anticoagulation therapy solely based on fall risk. Hence, discontinuation of anticoagulation should be a patient-specific decision that carefully considers the balance between the risk of traumatic brain injury (TBI) and the benefits of anticoagulation therapy. Factors such as age, frailty, and the type of anticoagulant should all be taken into account. Clinical judgment and selective CT imaging can help balance patient safety with healthcare costs.

Conclusion

The incidence of adverse outcomes following head injury in patients on anticoagulant therapy is 3.1% and relatively low. Careful decision-making regarding the discontinuation of anticoagulation therapy, informed by patient background and presentation and selective CT imaging, is essential to manage risks effectively and optimize healthcare resource utilization.

## Introduction

Falls and fractures represent a significant health challenge internationally, with individuals aged 65 and above experiencing the highest risk [1]. Historically within clinical practice, a fall while taking anticoagulation was thought to be of high significance and therefore investigated regardless of symptoms or signs adhering to current best practice guidelines [2]. In reference to NICE guidelines for the management of head injuries, a computed tomography (CT) brain scan (non-contrast) is recommended for patients on anticoagulants or antiplatelet therapy who present with a head injury, critical for detecting significant brain injuries within a specific timeframe, regardless of the Glasgow Coma Scale (GCS) level or focal neurological findings.

According to NHS England, data analysis up until November 2023 showed that approximately 1.5 million people in England are living with atrial fibrillation (AF) [3], which is responsible for one in five strokes, and consequently, around 0.5 million people with an increased risk of stroke have been prescribed direct oral anticoagulants (DOACs). Historically, vitamin K antagonists, such as warfarin, were the predominant class of anticoagulants used to prevent or treat thromboembolic events. However, more recently, DOACs have now increasingly supplanted these traditional agents, mainly due to their advantages in ease of use and reduced monitoring requirements [4]. According to the 2021/2022 Pharmacy Quality Scheme (PQS) community pharmacy oral anticoagulant safety audit, DOACs, including Apixaban and Rivaroxaban, are now the most frequently prescribed anticoagulants in the UK [5].

Despite their well-established benefits, the use of anticoagulants is often tempered amongst the older population with concerns regarding the risk of falls and subsequent injury. To this effect, the discontinuation of anticoagulant treatment has often been observed in clinical practice [6], yet according to current publications, there is a lack of significant evidence that quantifies this increased bleeding risk associated with anticoagulation and falls [7,8].

NICE guideline 2021 states that anticoagulation should not be withheld solely because of a person's age or their risk of falling [9].

This study aims to critically assess the incidence of significant brain injuries or intracranial haemorrhages in patients undergoing anticoagulation therapy and to generate comparisons with existing studies [10,11]

By examining the risk associated with TBI in this population, we seek to understand the impact of different anticoagulation therapies and the effectiveness of current management strategies. We hope this will aid clinical practice with more accurate risk/benefit assessments of anticoagulation in the elderly population with supportive data.

Aim

To evaluate the incidence of traumatic brain injury (TBI) following a fall in patients taking anticoagulant therapy, with a sub-analysis comparing the incidence of TBI between the medication groups. Furthermore, the study aims to evaluate the radiological investigation load and general economic impact on the National Health Service (NHS).

## Materials and methods

Study design and setting

This study was a retrospective, observational cohort study conducted at Queen Alexandra Hospital, Portsmouth Hospitals University NHS Trust, UK. The hospital serves a population of approximately 675,000 people, primarily older adults, making it an ideal setting for studying the impact of anticoagulants in a population at high risk for falls. The study period was from November 2023 to May 2024, during which time data on patient demographics, anticoagulant use, clinical outcomes, and CT scan results were collected and analysed.

Population and sample size

Study Population

The study focused on adults aged 18 years and older who presented to the hospital following a fall while on anticoagulant therapy. The anticoagulants of interest included vitamin K antagonists (e.g., Warfarin), direct oral anticoagulants (DOACs) such as Apixaban, Rivaroxaban, Edoxaban, and Dabigatran, as well as low molecular weight heparin (LMWH).

Sample Size

A total of 3,468 CT brain scan requests were screened during the study period. Eight hundred and one patients met the inclusion criteria, forming the final sample size. The sample size was determined based on all patients who met the criteria during the specified period. No formal preliminary sample size calculation was conducted, as the study aimed to include all eligible cases during the data collection timeframe.

Sampling Technique

The study used convenience sampling, where all eligible patients presenting during the study period who fulfilled the inclusion criteria were included. This non-probabilistic approach was appropriate given the retrospective nature of the study, which relied on existing clinical data from hospital records and radiology requests.

Inclusion and exclusion criteria

Inclusion Criteria

Patients aged 18 and older who were on anticoagulant therapy (DOACs, Warfarin, LMWH, or heparin) with or without additional antiplatelet medication at the time of their fall and who subsequently underwent a CT head scan, irrespective of Glasgow Coma Scale (GCS) score or neurological symptoms. This criterion aligns with current NICE guidance, which recommends head CT scans for anticoagulated patients following any head injury [2].

Exclusion Criteria

Patients not on anticoagulant therapy; patients taking antiplatelet therapy (e.g., aspirin, clopidogrel) without anticoagulants; and patients who suffer a head injury with an alternative mechanism, e.g., a road traffic accident or blunt trauma, but not from a fall.

The criteria were designed to focus on the risk of traumatic brain injury (TBI) within a specified cohort of anticoagulated patients, as these individuals are historically considered high-risk due to potential bleeding complications.

Data collection

Data were extracted from the hospital’s electronic health records (EHR) and radiology information systems. Relevant information included patient demographics and clinical data, including age, GCS at presentation, clinical frailty score (CFS), and type of anticoagulant (DOACs, Warfarin, LMWH).

Imaging Results

Result of CT head scans, including evidence of intracranial haemorrhage (ICH) or other traumatic brain injuries. (All radiological reports were reviewed by a consultant radiologist to ensure accuracy.)

Outcomes

Mortality (up to six months after presentation), any surgical intervention.

Data Management and Confidentiality

Data were anonymised before analysis, with each patient assigned a unique identifier. All data were stored on secure hospital servers, accessible only to authorised personnel in accordance with hospital information and governance policies and the UK Data Protection Act 2018.

Ethics and Data Security

Personal identifiers were removed, and data were stored on password-protected servers in compliance with the UK Data Protection Act. Registered and approved by the Clinical Audit Department, Portsmouth Hospitals University NHS Trust. Reg ID: 6009.

Data analysis

All statistical analyses were performed using IBM Corp. Released 2021. IBM SPSS Statistics for Windows, Version 28.0. Armonk, NY: IBM Corp. The following statistical methods were applied:

*Descriptive Statistics* 

Continuous variables, such as age and frailty score, and categorical variables, including the type of anticoagulant and GCS, were presented as frequencies and percentages.

Comparative Analysis

Differences in demographic and clinical characteristics between patients with and without TBI were evaluated using the chi-square test.

Multivariate Logistic Regression

Multivariate logistic regression was used to assess the independent predictors of TBI. The dependent variable was the presence or absence of TBI, and the independent variables include age (continuous or categorised <65 vs. ≥65), type of anticoagulation therapy (DOAC, Warfarin), GCS (≤14 vs. 15), and clinical frailty score.

Results were reported as odds ratios (OR) with corresponding 95% confidence intervals (CI). A p-value of <0.05 was considered statistically significant.

Quality Assurance

Radiological review: All CT scans were interpreted by senior radiologists. To ensure consistency, a random sample of 10% of scans was independently reviewed by a second radiologist.

Data validation: Double entry was used to minimise data entry errors, with two independent researchers cross-checking a random subset of 50 patient records.

Bias control: To reduce potential selection bias, the inclusion of all eligible patients within the study period ensured comprehensive capture of relevant cases.

## Results

Between November 2023 and May 2024, 3468 patients underwent a CT head presenting with a history of fall and head injury, out of which 801 were on anticoagulation.

Descriptive analysis

Age

The mean age of the study cohort was 83.12 years.

**Figure 1:**
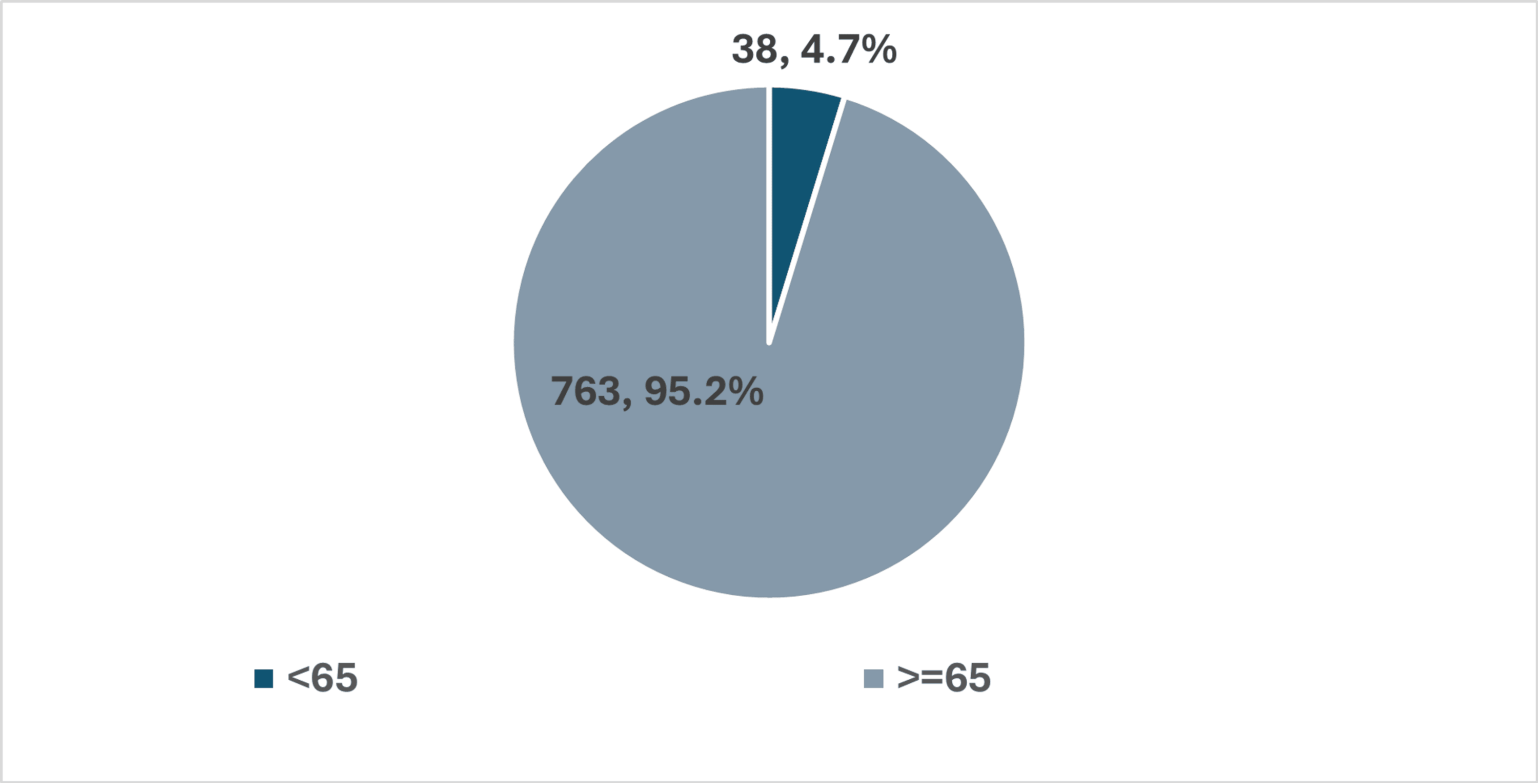
Age distribution of patients This figure displays the age distribution of patients who experienced falls while on anticoagulant therapy (n = 801). Comparison groups: Patients aged < 65 years vs. patients aged ≥ 65 years. This figure presents the age distribution of patients enrolled in the study (n = 801). The majority of patients (n = 763, 95.2%) are aged 65 years and older, while a minority (n = 38, 4.7%) are below the age of 65. p-value: Not applicable in this table, as it presents descriptive statistics rather than a comparative analysis.

Figure 1 categorises patients into two groups: those under 65 years of age, comprising 38 individuals (4.7%), and those aged 65 years and older, which includes 763 individuals (95.2%). This stark disparity underscores the study's focus on the elderly, who are particularly vulnerable to falls.

Clinical Frailty Score (CFS)

The CFS is an assessment tool that evaluates an individual’s overall health and functional status, particularly in older adults. CFS 4 and 5 represent a moderate level of frailty, indicating some vulnerability but generally independent living with occasional assistance. CFS 6 indicates a higher level of frailty, where individuals may require significant support in daily activities. CFS 7 reflects a very high level of frailty, with individuals likely needing extensive care and support.

**Figure 2:**
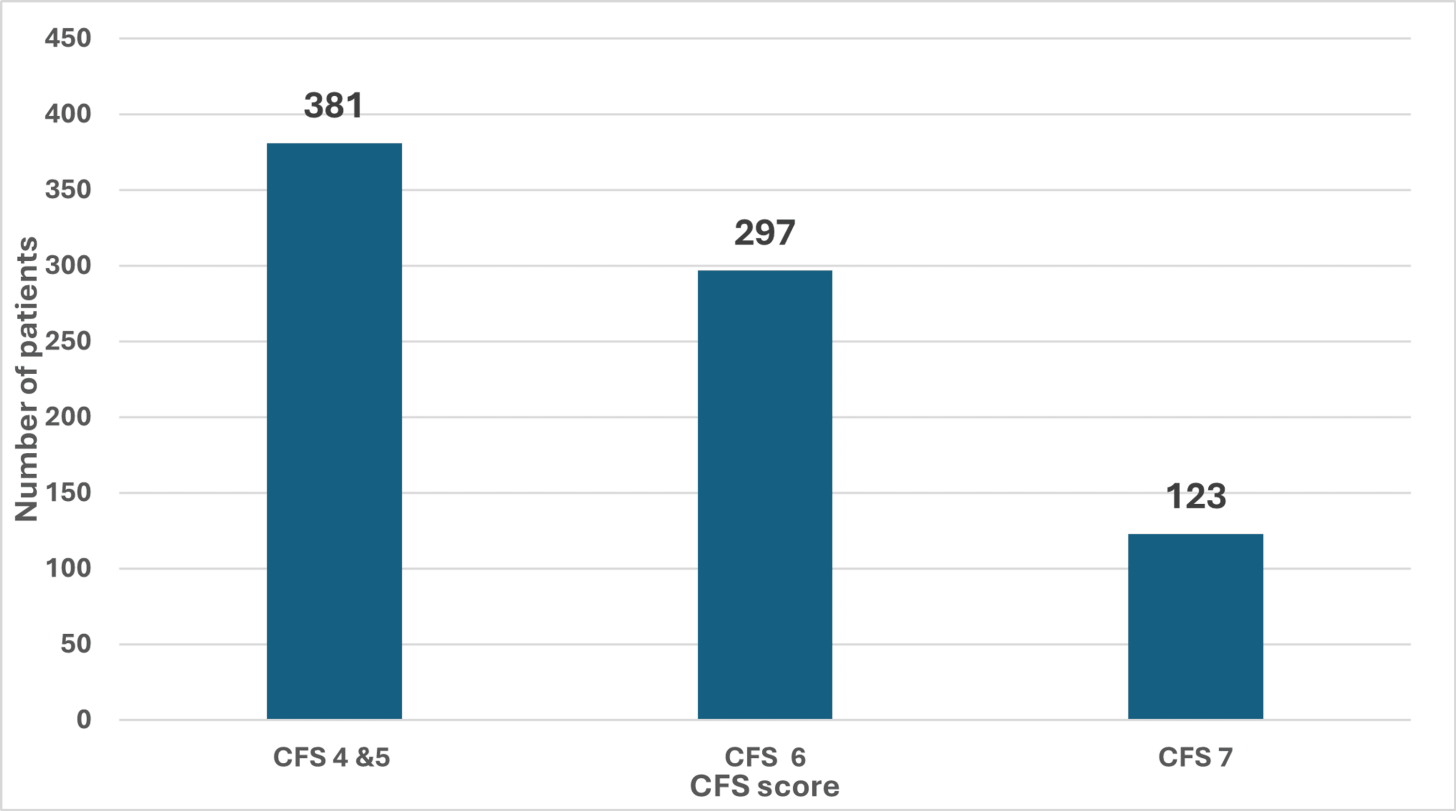
Performance status: clinical frailty score This figure categorises patients into three frailty levels: CFS 4 and 5, CFS 6, and CFS 7. This depicts the distribution of clinical frailty scores among our cohort of patients. The distribution of CFS reveals that nearly half of the patients, 381 (47.57%) out of 801, had a CFS 4 or 5, indicating moderate frailty. In contrast, 297 (37.08%) are classified as CFS 6, while 123 (15.36%) fall into the CFS 7 category, which signifies a very high degree of frailty.

Figure 2 categorises patients into three frailty levels: CFS 4 and 5, CFS 6, and CFS 7. This depicts the distribution of clinical frailty scores amongst our cohort of patients who experienced falls on anticoagulant therapy. The distribution of CFS reveals that nearly half of the patients, 381 (47.57%) out of 801, had a CFS 4 or 5, indicating moderate frailty. In contrast, 297 (37.08%) are classified as CFS 6, while 123 (15.36%) fall into the CFS 7 category, which signifies a very high degree of frailty. 420 (52.43%) patients had a CFS 6 and above, highlighting the significant levels of frailty associated with fall risk.

GCS at presentation

In a clinical assessment of patients' consciousness levels using the Glasgow Coma Scale (GCS), 610 patients (76.15%) presented with a GCS of 15, indicating full alertness and orientation. In contrast, 191 patients (23.85%) exhibited a GCS of 14 or lower, reflecting varying degrees of impaired consciousness. This distribution underscores the predominance of patients in a fully conscious state.

Anticoagulation: drug-wise distribution

**Figure 3:**
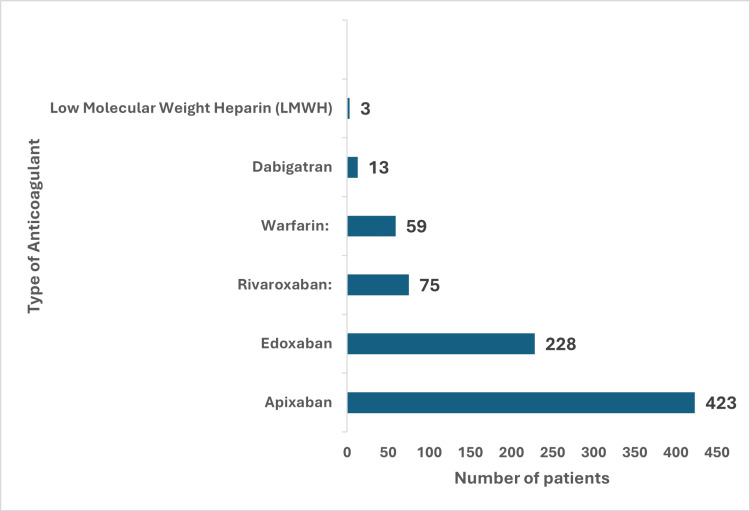
Distribution of anticoagulant medications prescribed to patients This figure summarises the distribution of anticoagulant medications among a total of 801 patients. The most commonly prescribed medication was Apixaban, which was administered to 423 patients, representing 52.81% of the total. Following Apixaban, Edoxaban was prescribed to 228 patients, accounting for 28.46% of the cohort. Rivaroxaban, Warfarin, and Dabigatran were prescribed to 75 (9.36%), 59 (7.37%), and 13 patients (1.62%), respectively. Low molecular weight heparin (LMWH) was the least prescribed, with only three patients (0.37%).

Figure 3 summarises drug-wise distribution among the patients. Among the 801 patients with fall-related head injuries who were on anticoagulation therapy, the majority, 423 (52.8%) were prescribed Apixaban, making it the most commonly used anticoagulant. Edoxaban was the second most frequently prescribed, accounting for 228 (28.46%) patients, followed by Rivaroxaban with 75 (9.3%) patients. Traditional anticoagulation with Warfarin was used by 59 (7.3%) patients, while DOAC Dabigatran was given to 13 (1.6%). Only three (0.37%) patients were on low molecular weight heparin (LMWH). This distribution demonstrates a strong preference for direct oral anticoagulants (DOACs), particularly Apixaban, with Warfarin being much less frequently prescribed.

Incidence of TBI

Out of the 801 patients receiving anticoagulation, 25 (3.12%) were found to have a confirmed CT diagnosis of acute haemorrhage. The 95% confidence interval is 1.91% to 4.33%.

Sub-analysis within this group showed nine patients (36%) had subdural hemomatoma (SDH), 11 patients (44%) had subarachnoid haemorrhage (SAH), four patients (16%) had intracerebral haemorrhage (ICH), and one patient (4%) had both subdural and subarachnoid haemorrhage.

Comparative analysis

Comparative Analysis of Anticoagulation Distribution and TBI (Traumatic Brain Injury)

Table 1 summarises the distribution of anticoagulants among the 801 patients and the corresponding incidence of TBI. Apixaban was the most commonly prescribed anticoagulant and had a TBI incidence of 2.8%, which served as the reference group for comparisons. Edoxaban (228 patients, 28.46%) and Rivaroxaban (75 patients, 9.36%) both showed TBI incidences of 2.6%, with no significant difference compared with Apixaban (p = 0.85 and p = 0.88, respectively). Warfarin, prescribed to 59 patients (7.37%), showed a significantly higher incidence of TBI (6.7%) and a p-value of 0.02, indicating a statistically significant difference compared to Apixaban. Similarly, Dabigatran, used by 13 patients (1.62%), had a TBI of 7.6%, and a p-value of 0.01, reflecting a significant difference. LMWH was used by three patients (0.37%); no adverse outcome data were available. This analysis highlights that Warfarin and Dabigatran are associated with a statistically significant higher risk of TBI compared with Apixaban, while the difference in risk between the DOACs (Edoxaban and Rivaroxaban) and Apixaban was not statistically significant.

**Table 1:** Anticoagulation therapy distribution and incidence of adverse outcomes (TBI) with p-values This table shows the distribution of anticoagulant use among 801 patients and their associated incidence of adverse outcomes (TBI following a fall). Statistical comparisons were made using the Apixaban group as the reference. The p-values indicate the level of significance when comparing other anticoagulants to the reference group. p < 0.05 (*): Statistically significant difference compared to Apixaban. p > 0.05 (ns): No significant difference compared to Apixaban. This analysis highlights that Warfarin and Dabigatran are associated with a statistically significant higher risk of TBI compared with Apixaban, while the difference in risk between the DOACs (Edoxaban and Rivaroxaban) and Apixaban was not statistically significant.

Anticoagulant	Number of Patients (%)	Incidence of Adverse Outcome (TBI)	p-value
Apixaban	423 (52.81%)	2.8%	Reference group
Edoxaban	228 (28.46%)	2.6%	0.85 (ns)
Rivaroxaban	75 (9.36%)	2.6%	0.88 (ns)
Warfarin	59 (7.37%)	6.7%	0.02 (*)
Dabigatran	13 (1.62%)	7.6%	0.01 (*)
LMWH	3 (0.37%)	N/A	N/A

Comparative Analysis of Age and Traumatic Brain Injury (TBI)

Table 2 shows the comparative analysis of age and TBI. Among the 801 patients included in the study, those aged 65 years and older represented the vast majority (763 patients, 95.3%), while only 38 patients (4.7%) were under 65 years old. Of the older adults, 25/763, or 3.3%, were diagnosed with TBI following a fall, whereas no cases of TBI were reported in the younger cohort. This disparity highlights a marked difference in TBI incidence associated with age, underscoring that older adults are at a significantly greater risk for TBI following a fall confirmed through statistical analysis, p-value <0.05, indicating that age is a significant predictor of TBI in patients on anticoagulant therapy.

**Table 2:** Comparative analysis of age and traumatic brain injury (TBI) This table presents the comparative analysis of the incidence of traumatic brain injury (TBI) based on age groups in patients on anticoagulant therapy following falls. It includes the total number of patients in each age group, the number of patients diagnosed with TBI, and the corresponding percentage of TBI cases within each age group. *The p-value indicates the statistical significance of the findings, highlighting that individuals aged 65 years and older are at a significantly higher risk of TBI compared to those under 65 years. This underscores the importance of age as a predictor of TBI risk in anticoagulated patients.

Age Group	Total Patients (n)	Patients with TBI (n)	Percentage of TBI (%)	p-value
< 65 years	38	0	0%	Reference
≥ 65 years	763	25	3.3%	< 0.05*

Comparative Analysis of Clinical Frailty Score (CFS) and Traumatic Brain Injury (TBI)

Table 3 shows a comparative analysis of the CFS in relation to the incidence of TBI. Among the 801 patients studied, the distribution of CFS scores indicated that 381 patients (47.57%) were classified as CFS 4 and 5 (indicating moderate frailty), 297 patients (37.08%) had a CFS score of 6 (high frailty), and 123 patients (15.36%) fell into the CFS 7 category (very high frailty). Of the patients with TBI, 10 (2.6%) were in the CFS 4 and 5 category, 9 (3.0%) were in the CFS 6 category, and 6 (4.9%) had a CFS score of 7. The analysis revealed a statistically significant increase in the incidence of TBI among patients with higher CFS, particularly in those with CFS 7 (p-value <0.001). This emphasises the concomitant increase in clinical frailty and the risk of sustaining a TBI in patients on anticoagulant therapy.

**Table 3:** Comparative analysis of clinical frailty score (CFS) and traumatic brain injury (TBI) This table presents the comparative analysis of the Clinical Frailty Score (CFS) and the incidence of traumatic brain injury (TBI) in patients on anticoagulant therapy following falls. It includes the total number of patients within each CFS category, the number of patients diagnosed with TBI, and the corresponding percentage of TBI cases for each frailty level. The p-value indicates the statistical significance of the findings, demonstrating that higher levels of clinical frailty are associated with an increased risk of TBI. This underscores the importance of assessing frailty in patients on anticoagulation therapy, particularly when considering the risk of falls and subsequent injuries.

Clinical Frailty Score (CFS)	Total Patients (n)	Patients with TBI (n)	Percentage of TBI (%)	p-value
CFS 4 & 5	381	10	2.6%	Reference
CFS 6	297	9	3.0%	< 0.01
CFS 7	123	6	4.9%	< 0.001

Comparative Analysis of Glasgow Coma Scale (GCS) and Traumatic Brain Injury (TBI)

Table 4 shows an analysis of GCS in relation to the incidence of TBI and highlights a significant correlation between altered consciousness and TBI risk. Among the 801 patients, 610 patients (76.15%) presented with a GCS score of 15, indicating full alertness and orientation, while 191 patients (23.85%) had a GCS score of 14 or lower, reflecting varying degrees of impaired consciousness. The findings revealed that of the patients with a GCS of ≤14, a notable proportion were diagnosed with TBI, indicating a significant increase in risk. In contrast, no TBI cases were found among patients with a GCS score of 15. The statistical analysis demonstrated a highly significant difference (p-value <0.001), emphasising that patients with a reduced GCS are at higher risk of TBI, highlighting the importance of immediate neurological assessment in this population.

**Table 4:** Comparative analysis of the Glasgow Coma Scale (GCS) and traumatic brain injury (TBI) This table presents the comparative analysis of Glasgow Coma Scale (GCS) scores and the incidence of traumatic brain injury (TBI) in patients on anticoagulant therapy following falls. It includes the total number of patients within each GCS category, the number of patients diagnosed with TBI, and the corresponding percentage of TBI cases for each GCS score. The p-value indicates the statistical significance of the findings, demonstrating that patients with GCS scores of ≤14 (indicative of altered consciousness) have a significantly higher incidence of TBI compared to those with a GCS score of 15 (fully alert). This underscores the critical importance of GCS assessment in identifying patients at elevated risk for TBI in this vulnerable population.

GCS Score	Total Patients (n)	Patients with TBI (n)	Percentage of TBI (%)	p-value
GCS 15	610	0	0	Reference
GCS ≤14	191	25	13.09	< 0.001

Logistic regression analysis

Table 5 summarises the results of a multivariate logistic regression analysis conducted to identify independent predictors of traumatic brain injury (TBI) among patients on anticoagulant therapy. Each predictor is associated with its odds ratio (OR), 95% confidence interval (CI), and p-value, providing insights into the strength and significance of these relationships.

**Table 5:** Independent predictors of traumatic brain injury (TBI) Odds ratios for predictors of outcome in patients receiving anticoagulation therapy. This table presents the odds ratios (OR) for various predictors associated with a specific outcome among patients on anticoagulation therapy. The odds ratios indicate the likelihood of the outcome occurring in relation to each predictor. The 95% confidence intervals (CI) provide a range in which the true odds ratio is expected to lie, while the p-values indicate the statistical significance of the results. An OR greater than one suggests an increased likelihood of the outcome associated with the predictor. The reference group for comparison is indicated for each predictor. Statistically significant p-values are (p < 0.05).

Predictor	Odds Ratio (OR)	95% Confidence Interval (CI)	p-value
Age ≥ 65	1.95	1.11 – 3.46	0.02
Warfarin vs. DOACs	3.56	1.62 – 7.83	0.01
GCS ≤ 14	5.12	2.40 – 10.91	< 0.001
CFS (6)	2.45	1.12 – 5.38	0.03
CFS (7)	2.70	1.30 – 5.60	0.008

Age is a significant predictor, with patients aged 65 years and older being nearly twice as likely to experience TBI compared to younger individuals (OR = 1.95, 95% CI: 1.11 - 3.46, p = 0.02). This finding highlights the heightened risk of TBI in older adults. Additionally, patients prescribed Warfarin have more than three times the odds of sustaining a TBI compared to those on DOACs (OR 3.56, 95% CI: 1.62 - 7.83, p = 0.01). This suggests that the type of anticoagulant plays a critical role in TBI risk, with Warfarin users exhibiting a greater vulnerability.

The analysis also reveals that a Glasgow Coma Scale (GCS) score of 14 or lower significantly increases the odds of TBI (OR 5.12, 95% CI: 2.40 - 10.91, p < 0.001). This indicates that patients with altered consciousness are over five times more likely to sustain a TBI compared to those with a GCS of 15. Furthermore, patients with a CFS of 6 (OR 2.45, 95% CI: 1.12 to 5.38, p = 0.03), indicating higher levels of frailty are associated with an increased risk of TBI, and those patients with a CFS of 7 have an even higher risk (OR 2.70, 95% CI: 1.30 - 5.60, p = 0.008), reinforcing the notion that greater frailty corresponds with a heightened risk of traumatic injuries. Collectively, these findings emphasise the multifaceted nature of TBI risk in anticoagulated patients, driven by factors including age, medication type, consciousness levels, and frailty status.

Management and six-month mortality

All 25 patients with acute haemorrhage on initial CT head were conservatively managed following consultation with the regional tertiary neurosurgical centre based in Southampton, UK.2 of the 25 patients died within six months of their acute haemorrhage.

## Discussion

In line with the findings of this study, the risk of TBI post-fall while on anticoagulation is relatively low (3.1%, which is comparable with existing published studies [7].

DOACs were the most frequently used anticoagulants in this study group. Medication analysis relating to adverse outcomes revealed that vitamin K antagonists were associated with a higher incidence of TBI compared with DOAC users. This finding suggests a potential advantage of DOACs in reducing the risk of TBI in older adults, highlighting the need for careful consideration when prescribing anticoagulation therapy in older adults. As the prevalence of anticoagulant use continues to rise, understanding the differential risks associated with various anticoagulants can guide clinical decisions and patient management strategies.

The statistical analysis conducted in this study provides compelling evidence for the risk factors associated with TBI. Notably, age emerged as a significant risk factor, with patients aged 65 and older exhibiting a markedly higher incidence of TBI compared to their younger counterparts. Additionally, frailty, as measured by the Clinical Frailty Score (CFS), significantly correlated with TBI risk, particularly in patients with scores of 6 or higher. This statistical association reinforces the importance of frailty assessments in clinical settings, as identifying frail individuals can aid in the development of targeted interventions to prevent falls and subsequent injuries. Furthermore, the Glasgow Coma Scale (GCS) scores demonstrated a significant relationship with TBI incidence, with lower GCS scores indicating a heightened risk of brain injury. These findings underscore the necessity of neurological assessments in patients presenting after falls, particularly those on anticoagulation therapy.

Considering the risk/benefit of anticoagulation, a well-considered decision must be made before the discontinuation of anticoagulation therapy solely because of falls, given the low incidence of adverse outcomes. This contrasts with the risk and incidence of stroke associated with AF and the significant implications of this diagnosis [12].

Hence, discontinuation of anticoagulation should be a patient-specific decision that carefully considers the balance between the risk of traumatic brain injury (TBI) and the benefits of anticoagulation therapy. Factors such as age, frailty, and the type of anticoagulant should all be taken into account. The findings of this study suggest that while direct oral anticoagulants (DOACs) like Apixaban may present a lower risk of TBI compared to traditional options like Warfarin, the decision to continue or discontinue anticoagulation due to falling risk should be individualised based on the patient's overall risk profile. This includes evaluating the necessity of anticoagulation for preventing thromboembolic events and weighing it against the potential for fall-related injuries. By personalising these decisions, clinicians can optimise care, minimising adverse outcomes while maximising therapeutic benefits for each patient.

Considering the economic impact and radiological workload, along with the risk of TBI identified in this study, clinical judgement based on presentation and selective CT imaging may offer significant benefits. Future research efforts should focus on establishing clear criteria for head CT scans following falls and validating risk assessment models for both positive and negative imaging outcomes. This approach aims to reduce unnecessary imaging, thereby lowering healthcare costs and minimising patient exposure to unwarranted radiation.

Study limitation 

The data were collected from a single healthcare system, which may limit the generalisability of the findings to other populations or healthcare settings. The number of patients using less common anticoagulants (e.g., Dabigatran, LMWH) is small, which may affect the reliability of the statistical analysis for these groups. Other factors, such as comorbidities, medication adherence, and fall severity, were not thoroughly addressed, potentially influencing the results.

## Conclusions

In conclusion, the incidence of traumatic brain injury (TBI) following falls in patients on anticoagulation therapy was relatively low at 3.1%, reinforcing the notion that anticoagulation should not be discontinued solely based on falls’ risk. This should be individualised, following an informed discussion with the patient or relatives culminating in patient-centered decision outcomes. Age, frailty, and anticoagulation type emerged as significant predictors of TBI, emphasising the importance of individualised patient assessments. The study highlights that DOACs, especially Apixaban, are associated with a lower risk of TBI compared to Vitamin K antagonists, suggesting a potential advantage in their use for older adults at risk of falls. Clinical decision-making, informed by the patient’s background, symptoms, and signs, supported by selective use of CT imaging, can assist with balancing patient safety and the requirement of mitigating healthcare costs.
